# DEPDC1在肺腺癌中高表达并促进肿瘤细胞增殖

**DOI:** 10.3779/j.issn.1009-3419.2021.103.06

**Published:** 2021-07-20

**Authors:** 健 沈, 萌萌 郗

**Affiliations:** 1 121001 锦州，锦州医科大学附属第三医院风湿免疫科 Department of Rheumatology and Immunology, The Third Affiliated Hospital of Jinzhou Medical University, 121001 Jinzhou, China; 2 121001 锦州，锦州市妇婴医院呼吸科 Department of Respiratory Medicine, Jinzhou Women and Infant Hospital, 121001 Jinzhou, China

**Keywords:** DEPDC1, 肺肿瘤, 预后, 生物标志物, 细胞增殖, DEPDC1, Lung neoplasms, Prognostis, Biomarker, Cell proliferation

## Abstract

**背景与目的:**

肺癌是目前世界范围内造成死亡人数最多的癌症之一，而肺腺癌是其主要分型。DEPDC1（DEP domain containing 1）被证实与多数肿瘤的发生发展密切相关，DEPDC1在肺腺癌中过表达已被初步证实，本研究旨在探究DEPDC1的表达与肺腺癌临床预后的关系，对DEPDC1作为肺腺癌潜在生物标志物和治疗靶点的可能性进行初步探讨。

**方法:**

利用生物信息学网站GEPIA搜集相关信息，在线分析DEPDC1表达与患者预后生存关系。收集本医院患者资料，对收集的样本进行免疫组化染色，并进行统计学分析。随后，体外培养肺腺癌系细胞，通过Western blot与逆转录定量聚合酶链反应（reverse transcription-quantitative polymerase chain reaction, RT-qPCR）验证敲低效率，再进行细胞增殖的实验。

**结果:**

DEPDC1在肺腺癌组织中表达显著高于癌旁正常组织，DEPDC1在肺腺癌组织中高表达，DEPDC1高表达与肺腺癌的肿瘤大小临床分期相关，敲低DEPDC1可抑制A549和H1975细胞增殖。

**结论:**

DEPDC1在肺腺癌的进展演变中扮演着重要角色，有望成为肺腺癌重要的治疗靶点和一个潜在的新的生物标志物。

## Abstract (en)

**Background and objective:**

Lung cancer is the leading cause of death worldwide, and lung adenocarcinoma is the main subtype of lung cancer. DEP domain-containing 1 (DEPDC1) has been proved to be closely related to the occurrence and development of most tumors, and the overexpression of DEPDC1 in lung adenocarcinoma has been preliminarily confirmed. This study aims to explore the relationship between the expression of DEPDC1 and the clinical prognosis of lung adenocarcinoma, and to preliminarily explore the possibility of DEPDC1 as a potential biomarker and therapeutic target of lung adenocarcinoma.

**Methods:**

The bioinformatics website GEPIA database was used to collect relevant information, and the prognostic was analyzed online. Patient data were collected for statistical analysis, and immunohistochemical staining was performed on the collected samples. Subsequently, lung adenocarcinoma cells were cultured *in vitro*, and the knockout efficiency was verified by Western blot and reverse transcription-quantitative polymerase chain reaction (RT-qPCR), and cell proliferation experiments were performed.

**Results:**

The expression of DEPDC1 in lung adenocarcinoma tissues is significantly higher than that in adjacent normal tissues. The high expression of DEPDC1 is correlated with the tumor size and clinical stage of lung adenocarcinoma and knocking down DEPDC1 inhibits the proliferation of A549 and H1975 cells.

**Conclusion:**

DEPDC1 plays an important role in the progression and evolution of lung adenocarcinoma. And it is expected to become an important therapeutic target and a potential new biomarker for lung adenocarcinoma.

肺癌是中国乃至世界发病率和死亡人数最高的肿瘤之一，根据美国癌症协会的数据^[1-3]^统计，近年来肺癌的发病和死亡人数仍在持续增加。而根据组织学特征，肺癌分为两种亚型：小细胞肺癌（small cell lung cancer, SCLC）和非小细胞肺癌（non-small cell lung cancer, NSCLC）^[4]^。NSCLC进一步分为3种主要的组织学亚型：肺腺癌（lung adenocarcinoma, LUAD）、肺鳞状细胞癌和大细胞癌^[5]^。目前针对肺癌的治疗包括靶向治疗和免疫疗法与传统手术，放疗和化学疗法相结合，为提高患者的生存率做出了巨大贡献。然而，由于诊断的局限性，大多数NSCLC患者已经处于晚期^[6]^，其5年生存率很低。因此，为了提高患者的预后和生存率，发现一种新的生物标志物，提高肺腺癌的诊断率，寻找到新的药物治疗靶点已迫在眉睫。

DEPDC1（DEP domain containing 1）是一种蛋白质编码的基因，除睾丸外，正常人体组织中均未检测到它的表达^[7]^。DEPDC1已经被证实与多个肿瘤的发生密切相关，例如前列腺癌^[8]^、乳腺癌^[9]^、胃癌^[10]^、结直肠癌^[11]^、肝细胞癌^[12]^。并且在这些肿瘤中高度表达，并且与临床预后不良密切相关。以上的研究均表明，DEPDC1在大多数肿瘤中过表达，具有成为生物标志物和治疗靶点的潜力。DEPDC1在肺腺癌中过表达也被初步证实，有研究^[13]^证实靶向DEPDC1会激活核因子κB（nuclear factor kappa-B, NF-κB）信号通路介导肺腺癌细胞的凋亡。但是他们的研究并没有用免疫组化验证DEPDC1的组织表达水平，也没有进行动物实验，论证完整性欠缺，关于二者的机制仍需要进一步探讨。

本研究旨在通过探究肺腺癌组织与癌旁正常组织中DEPDC1的表达量及其临床预后的差异，并且通过体外实验去进一步探究敲低DEPDC1对癌细胞增殖的影响。这将有助于我们进一步理解DEPDC1在肺腺癌的发生发展中所发挥的重要作用，为探索肺腺癌的生物标记物提供新的思路。

## 1 材料与方法

### 1.1 样本资料

本研究共纳入锦州医科大学附属第三医院收治的65例肺腺癌手术患者，所有标本组织均经术后病理证实。根据美国癌症联合委员会（American Joint Committee on Cancer, AJCC）肿瘤原发灶-淋巴结-转移（tumor-node-metastasis, TNM）2010分类系统评估肿瘤分期。

本研究以1964年赫尔辛基宣言和随后所有修订案中规定的道德标准为基础。所有案例的数据和标本采集工作均获得患者本人的知情同意，并签署知情同意书。

### 1.2 试剂

DEPDC1抗体（ab197246, IHC 1:200, WB 1:1, 000），DEPDC1二抗（PV6000），Trizol试剂（Thermo Fisher Scientific，美国马萨诸塞州沃尔瑟姆），SuperSilencing™ shRNA质粒表达载体套装（吉玛公司，中国，上海C01001），Cell Counting Kit-8（CCK-8, Dojindo Molecular Technologies, Gaithersburg, MD）。

### 1.3 免疫组织化学染色及结果评定

肿瘤组织和邻近的正常组织石蜡切片在我院采集。从石蜡块上切下切片（4 μm），进行脱蜡和抗原修复后，与DEPDC1抗体（ab197246, Abcam plc, Cambridge, UK. 1:200）一起在4 oC下孵育过夜。然后，在室温下应用通用性（小鼠/兔聚合物法检测系统）PV-6000二步法检测试剂盒（PV-6000，中杉金桥生物有限公司，北京）进行二抗染色30 min。使用DAB显色试剂盒（ZLI-9018，优宁维生物科技股份有限公司，上海）进行后续染色。应用苏木素（G1080，百泰克生物科技有限公司，北京）进行最后的细胞核染色，后续进行梯度酒精脱水和二甲苯透明，中性树胶封片，显微镜下观察和照相采图。染色强度评分如下：无染色为0分；弱染色为1分；中高度染色为2分。染色阳性细胞的百分比分类如下：无染色，0分：1%-24%的染色细胞；1分：25%-50%；2分：51%-84%；3分：85%-100%。由两名病理学家评估免疫组织化学（immunohistochemistry, IHC）染色。最终分数是通过将比例和强度相乘得出的。根据最终分数的分布，我们将DEPDC1的表达分为高表达组（3分-8分）和低表达组（0分-3分）。

### 1.4 细胞培养

人肺腺癌细胞株A549和H1975来自中国科学院细胞库（中国上海）。在37 oC、95%空气和5%CO_2_的培养箱中，将细胞维持在10%胎牛血清（FBS, Gibco）的RPMI-1640（Gibco）中。以经携带shRNA质粒处理的细胞为shRNA组，以携带无效靶点的shRNA的细胞为对照组。

### 1.5 稳定的敲低细胞系

为了建立稳定敲低DEPDC1的细胞系，我们选择了有效的shRNA序列，shRNA的序列是5'-AAACATCGCTGTCGTTTCAAGAG-3'。选用SuperSilencingTM shRNA质粒表达载体套装，使用pGPH1/Neo载体，构建质粒经大肠杆菌扩增后使用lipo3000转染，3 d后使用新霉素筛选。逆转录定量聚合酶链反应（reverse transcription-quantitative polymerase chain reaction, RT-qPCR）和Western blot用于检测两种细胞系的敲低效率。选择稳定敲低细胞并用于以后的实验。

### 1.6 RT-qPCR

按照制造商的说明，用Trizol试剂（Thermo Fisher Scientific，美国马萨诸塞州沃尔瑟姆）提取细胞的总RNA。通过cDNA逆转录试剂盒（Thermo Fisher Scientific，具体同上）将总RNA逆转录为cDNA。然后，进行实时定量PCR。DEPDC1引物（上海生工，中国）：DEPDC1-正向引物：5'-TTTTGGTCCTGAAGTTACAAGGC-3'和DEPDC1-反向引物：5'-TGGATACCTTCGTGGTAGAGTTT-3'。

### 1.7 Western blot

提取蛋白后，按照试剂盒说明书制胶，加样后，设置电压为60 V时间为15 min，随后设置为100 V，时间为110 min。取出SDS-PAGE胶，进行转膜。取出PVDF膜，经TBST溶液清洗，用10%脱脂牛奶进行封闭。TBST清洗3次，每次3 min后，将膜放入一抗中，4 oC摇床过夜。第二天室温下TBST清洗PVDF膜，每次10 min共3次后，加入二抗，摇床孵育1 h，再经TBST清洗3次，每次5 min后，显影。

### 1.8 细胞克隆形成实验

将A549和H1975细胞接种于6孔板中，每孔500个细胞。第2天用DMSO或BMP受体拮抗剂处理细胞2周。集落用Diff-Quick（IMEB Inc.San Marcos, CA）染色，计数每孔菌落总数。

### 1.9 细胞增殖试验

采用细胞计数试剂盒-8（cell counting kit-8, CCK-8）法，按照制造商说明书检测细胞增殖能力。每孔加入10 μL细胞计数试剂盒-8（CCK-8, Dojindo Molecular Technologies, Gaithersburg, MD）的溶液，将这些细胞放置于细胞培养箱中培养1 h。最后，使用酶标仪（Bio-Rad, Hercules, CA, USA）在450 nm处测量吸光度。

### 1.10 GEIPA书数据库

GEPIA数据库是一个在线生物信息数据库（http://gepia.cancer-pku.cn/detail.php?gene=DEPDC1），提供有关不同癌症的生物学信息分析。我们从TCGA数据库下载了LAUD患者的临床数据和mRNA表达数据。然后比较DEPDC1在肺腺癌组织和癌旁对照组的表达差异。分析了DEPDC1在LAUD患者的总生存期（overall survival, OS）和无病生存期（disease-free survival, DFS）中的预后价值。

### 1.11 统计分析

采用SPSS 26.0软件对数据进行分析，在定量分析时采用均数±标准差（Mean±SD）表示，同时使用*t*检验对两组进行统计比较。用χ^2^分析探讨临床病理特征与蛋白表达水平的关系。*P* < 0.05认为差异有统计学意义。

## 2 结果

### 2.1 DEPDC1在肺腺癌组织生物信息学分析

通过GEPIA数据库中在线分析了350个临床样本（其中包含483个肺腺癌临床样本和347个正常肺组织样本，http://gepia.cancer-pku.cn/detail.php?gene=DEPDC1）。结果显示DEPDC1的表达量在肺腺癌组织中明显高于正常肺组织（*P* < 0.05）（图 1A）。为了进一步探讨DEPDC1的表达与肺腺癌患者的预后之间的潜在关系，通过使用*Kaplan-Meier*绘制DEPDC1与肺腺癌患者预后的关系。结果显示，在DEPDC1表达量高的患者中，OS率和DFS率都比较低（图 1B），高表达组与低表达组的预后差异具有统计学意义（*P* < 0.05）。以上结果显示DEPDC1在肺腺癌组织中的表达水平高，并与肺腺癌的预后生存紧密相关。

**图 1:**
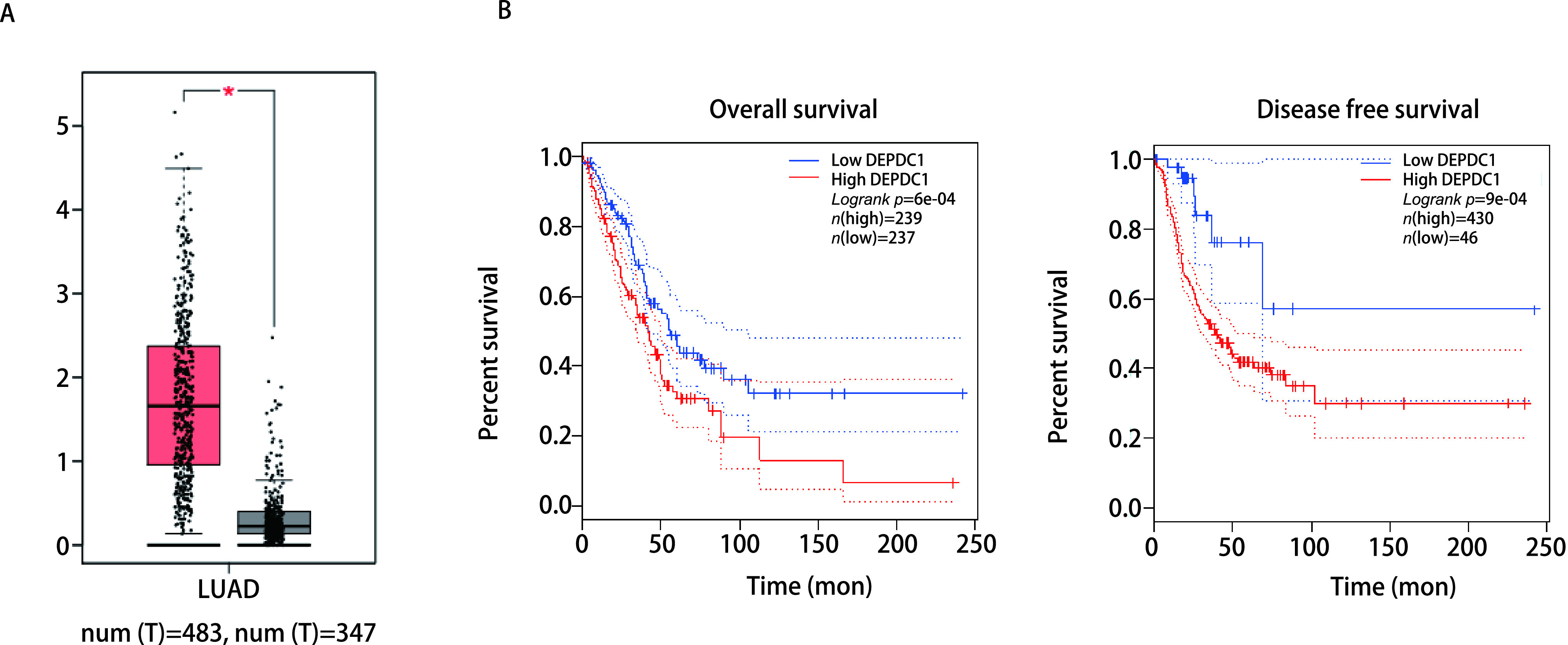
DEPDC1在肺腺癌组织中的生物信息学分析。A：肺腺癌组织与正常组织DEPDC1表达量对比；B：DEPDC1高低表达组总体生存率与无病生存率对比（*P* < 0.05）。 Bioinformatics analysis of DEPDC1 in lung adenocarcinoma tissue. A: Comparison of the expression level of DEPDC1 in lung adenocarcinoma tissues and normal tissues; B: Comparison of overall survival rate and disease-free survival rate between the high and low expression group of DEPDC1 (*P* < 0.05).

### 2.2 DEPDC1在肿瘤组织中的表达情况

通过GEPIA数据库在线分析了DEPDC1在肿瘤中的表达情况，结果显示，在大多数肿瘤的中的表达是阳性的，在甲状腺癌、头颈部鳞癌、尿路上皮癌、黑色素瘤中高度表达（https://www.proteinatlas.org/ENSG00000024526-DEPDC1/pathology）。而在淋巴瘤中未见明显表达。我们进一步探究了DEPDC1在结直肠癌、乳腺癌、前列腺癌、肺癌、肝癌中的表达情况，发现其在乳腺癌、结直肠癌中明显高表达，且发现DEPDC1主要表达在细胞核（图 2）。

**图 2:**
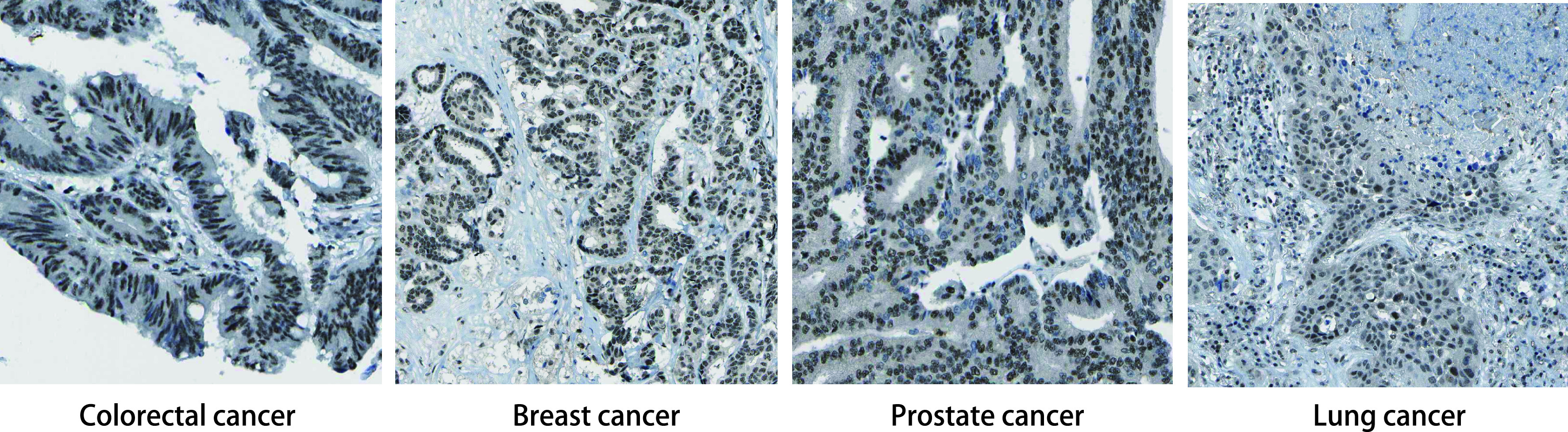
DEPDC1在多种肿瘤中的表达情况 Expression of DEPDC1 in a variety of tumors

### 2.3 DEPDC1在肺腺癌组织中高表达

为了探究DEPDC1在肺腺癌中的表达情况，我们运用免疫组织化学染色来检测65例肺腺癌组织中的DEPDC1的表达情况。结果显示，DEPDC1在肺腺癌组织中高度表达，并且DEPDC1主要定位于细胞核（图 3A）。根据染色的强度，我们人为地将肺腺癌患者分为高表达组和低表达组，用正常的肺组织来设立对照组，发现DEPDC1在癌旁的正常肺组织的表达量显著低于肺腺癌组织（图 3B）。

**图 3:**
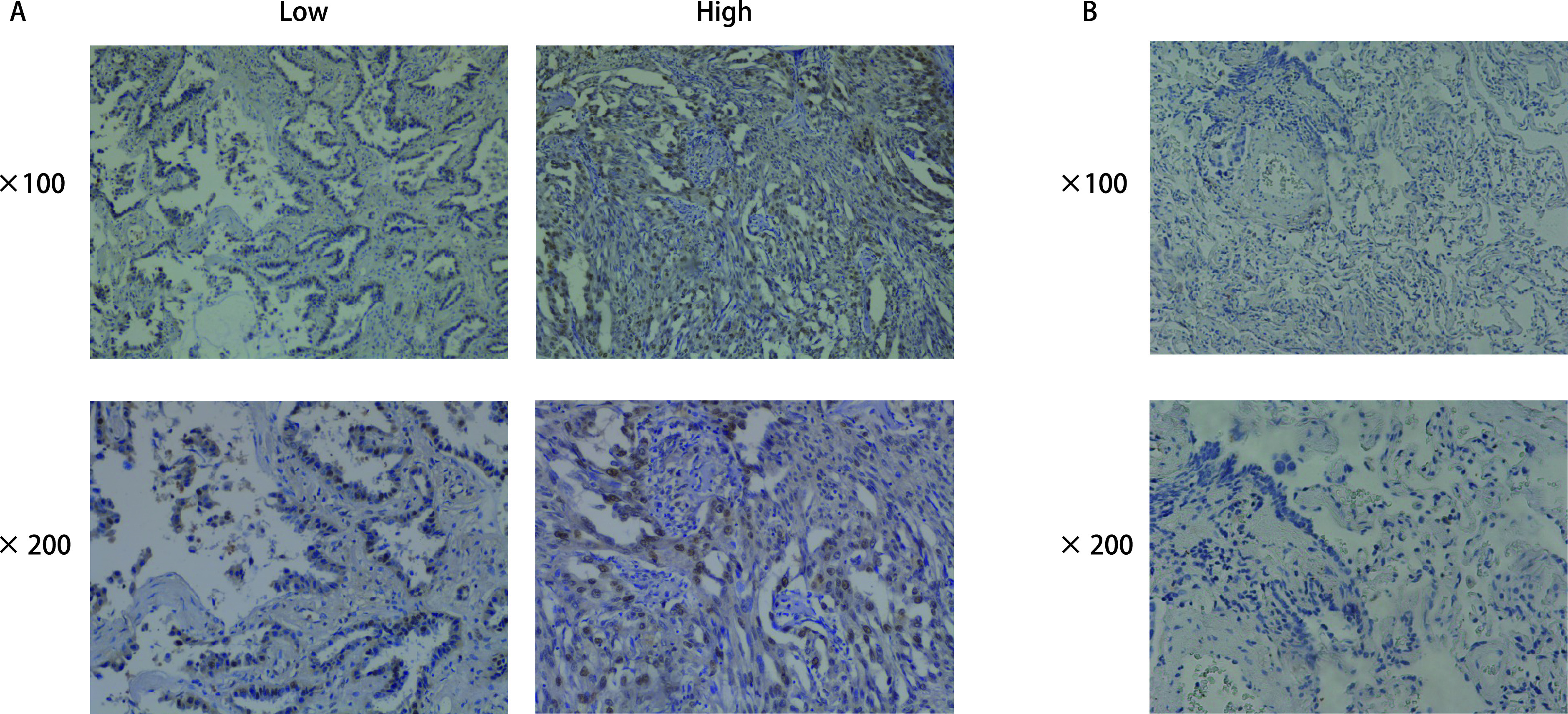
DEPDC1在肺腺癌组织中高表达。A：肺腺癌组织，分为高表达组与低表达组；B：癌旁正常组织。分别于×100与×200光镜下观察，肺腺癌组织染色强度显著高于癌旁组织。 DEPDC1 was highly expressed in lung adenocarcinoma tissues. A: lung adenocarcinoma tissue, divided into high and low expression group; B: It is normal tissue adjacent to the cancer. The staining intensity of lung adenocarcinoma tissues was significantly higher than that of adjacent tissues under ×100 and ×200 microscopes, respectively.

### 2.4 DEPDC1高表达与肺腺癌的肿瘤大小及临床分期相关

对上述的65例肺腺癌患者标本组织的DEPDC1表达量与病理参数的关系进行了统计学分析（表 1）。选取了性别、年龄、是否吸烟、肿瘤大小、分化和临床分期6个临床病理特征进行统计学分析，结果显示，DEPDC1与肿瘤大小（*P*=0.002）和临床分期（*P*=0.008）具有相关性，与性别、年龄、是否抽烟、肿瘤分化均无显著相关性（*P* > 0.05）。

**表 1:** DEPDC1的表达量与肿瘤临床特点的关系（*n*=65） Correlation of expression of DEPDC1 with clinical features of tumors (*n*=65)

Features	*n*	DEPDC1		*χ*^2^	*P*
		Low-expression (*n*=28)	High-expression (*n*=37)		
Age (yr)				0.565	0.452
< 55	36	17	19		
≥55	29	11	18		
Gender				0.293	0.588
Male	35	14	21		
Female	30	14	16		
Smoking				1.784	0.182
Yes	43	16	27		
No	22	12	10		
Tumor size (cm)				9.323	0.002
< 4	30	19	11		
≥4	35	9	26		
Tumor differentiation				0.744	0.388
Low	24	12	12		
High	41	16	25		
Clinical stage				7.116	0.008
Ⅰ	23	15	8		
Ⅱ-Ⅲ	42	13	29		
DEPDC1: DEP domain containing 1.					

### 2.5 敲低DEPDC1可抑制A549和H1975细胞增殖

通过RT-qPCR和Western blot检测DEPDC1在shRNA组和对照中的表达，以确认DEPDC1的敲低效率。shRNA组中的DEPDC1在mRNA和蛋白质水平上均得到显著抑制（图 4）。因此，在A549和H1975细胞系中成功构建了有效的DEPDC1敲低细胞模型。为了探索DEPDC1在A549和H1975细胞增殖中的作用，进行了细胞克隆实验和CCK-8实验。结果显示，敲低DEPDC1显著抑制了A549和H1975细胞增殖（图 5）。

**图 4:**
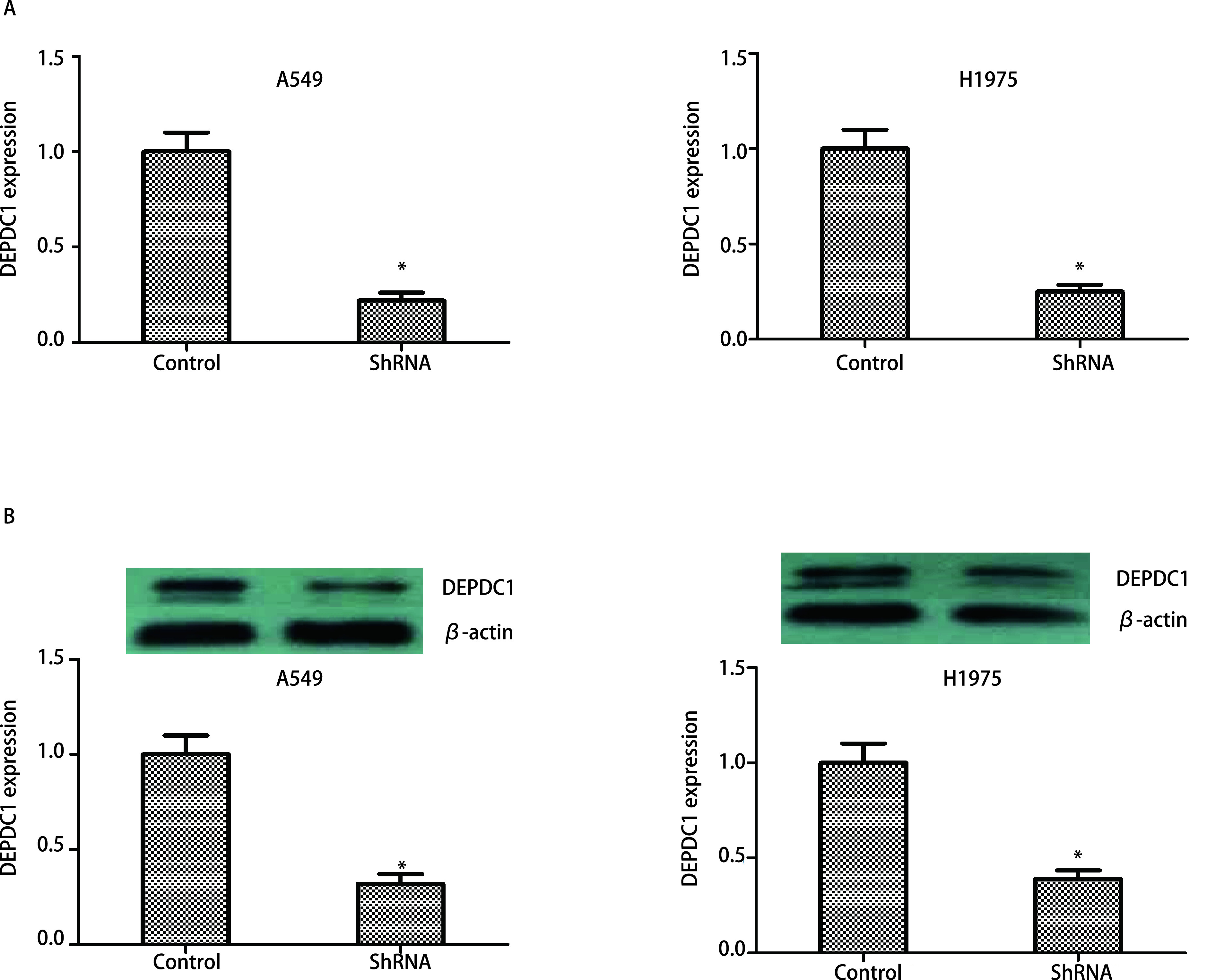
在A549和H1975细胞系中稳定敲低DEPDC1。A：使用RT-qPCR检测DEPDC1 mRNA的表达；B：使用Western blot检测DEPDC1蛋白表达。*P < 0.05。 Stably knock down DEPDC1 in A549 and H1975 cell lines. A: Detected the mRNA expression using RT-qPCR; B: Detected the protein expression using Western blot.*P < 0.05. RT-qPCR: reverse transcription-quantitative polymerase chain reaction.

**图 5:**
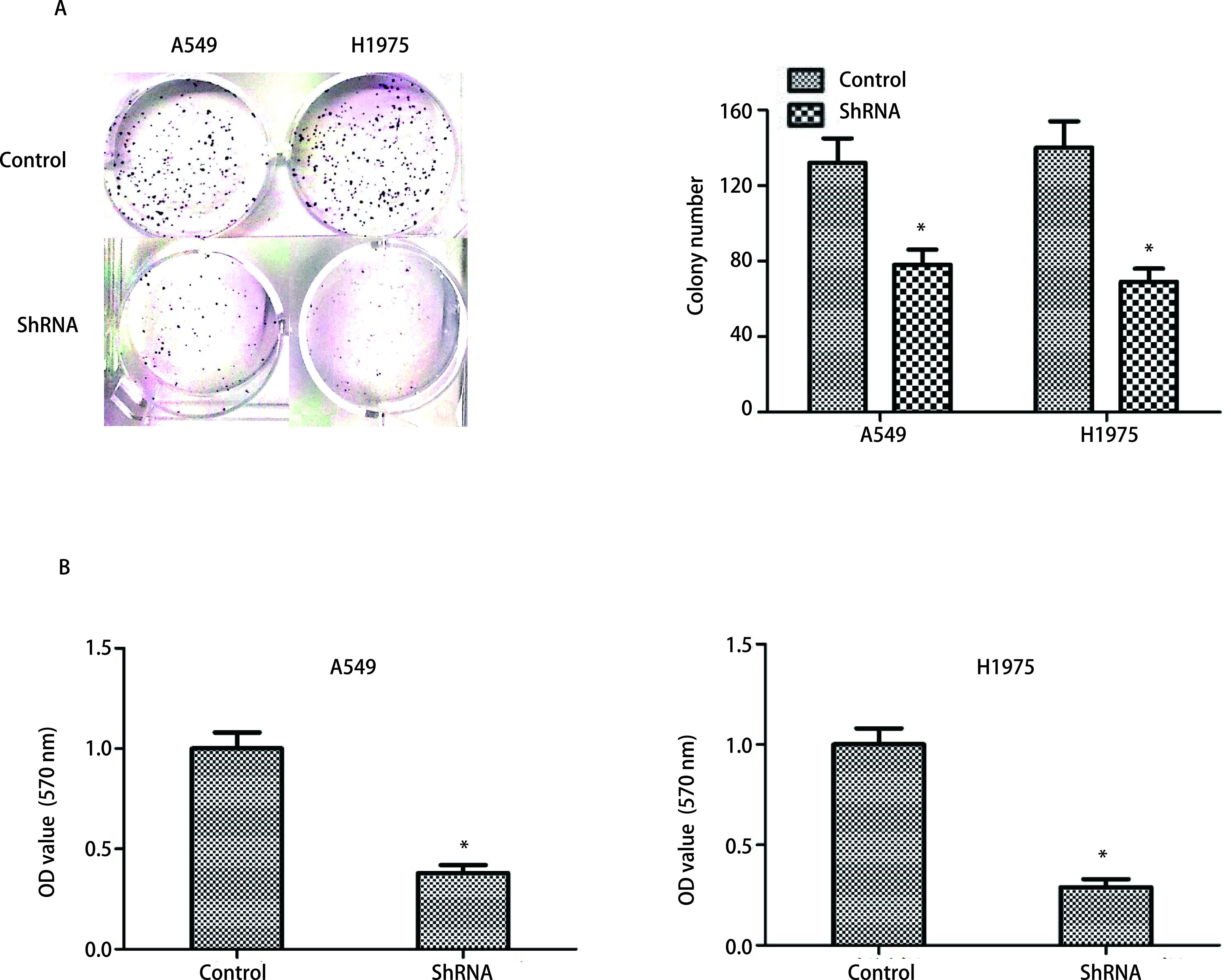
DEPDC1促进肿瘤细胞增殖。A：正常和敲低组A549和H1975细胞克隆实验；B：正常和敲低组A549和H1975 CCK-8实验。*P < 0.05。 DEPDC1 promotes the proliferation of tumor cells. A: Cell colony formation assay in shRNA and scramble cells of A549 and H1975; B: CCK-8 assay in shRNA and scramble cells of A549 and H1975. *P < 0.05.

## 3 讨论

肺癌已经对当今世界各地的人们造成了严重的健康威胁，发现肺癌新的治疗靶标和药物势在必行。部分患者可能仅有咳嗽和咳痰^[14]^的症状，常常易被断为呼吸道疾病、咽炎、气道过敏等，早期诊断和精确治疗是防治肺腺癌的有效手段。但是，肺腺癌的早期检测非常困难，早期的筛查方法是低剂量计算机断层扫描（computed tomography, CT）。肺腺癌的各种生物标志物已被用于临床诊断，如癌胚抗原（carcinoembryonic antigen, CEA）、癌抗原125（cancer antigen 125, CA125）等，这些标志物对肺腺癌^[15]^的早期诊断具有重要意义，但这些生物标志物仍不具备早期特异性地检测肺腺癌的能力。

DEPDC1在多数肿瘤中被证实高表达，Amisaki等^[12]^发现与正常肝脏相比，肝细胞癌患者的癌组织的DEPDC1上调，DEPDC1在肿瘤组织中的高表达与肿瘤进展和不良预后有关。Feng等^[16]^发现，与正常或非肿瘤组织相比，DEPDC1在鼻咽癌组织中的mRNA和蛋白水平均过表达，siRNA介导的DEPDC1缺失显著抑制了鼻咽癌细胞系CNE-1和HNE-1的增殖。Gong等^[10]^也发现了，与相邻正常胃组织相比，DEPDC1在胃腺癌组织中过表达，并且DEPDC1表达水平与癌症转移和分化显著相关。上述研究与我们所发现的“DEPDC1在肺腺癌中的高度表达与预后不良相关”的结论是一致的，我们通过对收集到的65例样本进行分析后，发现DEPDC1在癌组织中的表达量比正常肺组织显著增高，并且经过敲低DEPDC1后，癌细胞系克隆增殖明显受到抑制。

关于DEPDC1在肿瘤中的作用机制已有部分研究。Guo等^[17]^研究表明，DEPDC1通过CCL20/CCR6通路促进肝癌细胞增殖、侵袭和血管生成。Kikuchi等^[18]^发现了在小鼠胶质瘤模型中，DEPDC1在胶质瘤细胞系和组织中表达增加。小干扰RNA（siRNA）抑制内源性DEPDC1表达可通过NF-κB信号通路抑制胶质瘤细胞的活力，诱导细胞凋亡。上述研究均表明，DEPDC1在肿瘤发生发展中扮演了重要角色，而关于DEPDC1在肺腺癌中的作用机制，Wang等^[19]^研究表明DEPDC1在肺腺癌组织中上调，并且DEPDC1通过RAS-ERK1/2信号传导抑制A549、HCC827和H1993细胞的自噬。前文提到的Wang等^[19]^研究表明DEPDC1通过抑制A20表达来调节NF-κB活性，从而抑制A549细胞的凋亡。关于二者之间的具体作用机制有待我们进一步去探究。

综上所述，我们的研究初步证明了DEPDC1在肺腺癌中高表达与临床分期、肿瘤大小相关，并与OS存在显著相关性，故DEPDC1可能是肺腺癌的一项新的独立预后指标。我们的结果有助于进一步理解DEPDC1与肺腺癌之间的关系，鉴于二者的关系，通过深入的机制探究，DEPDC1有可能成为肺腺癌的一个潜在的生物标志物。但是我们的研究中所纳入的病例数量较少，可能存在一些统计误差。此外GEPIA数据库的样本数据也可能存在一定的误差。我们未能对其具体机制进行探究，这是我们下一步将去完成的工作。下一步我们将构建小鼠模型，并通过基因芯片、高通量测序等技术，以探究二者间具体作用机制。
